# Key Points in Air Pollution Meteorology

**DOI:** 10.3390/ijerph17228349

**Published:** 2020-11-11

**Authors:** Isidro A. Pérez, Mª Ángeles García, Mª Luisa Sánchez, Nuria Pardo, Beatriz Fernández-Duque

**Affiliations:** Department of Applied Physics, Faculty of Sciences, University of Valladolid, Paseo de Belén, 7, 47011 Valladolid, Spain; magperez@fa1.uva.es (M.Á.G.); mluisa.sanchez@uva.es (M.L.S.); npardo@fa1.uva.es (N.P.); beatriz.fernandez.duque@uva.es (B.F.-D.)

**Keywords:** particulate matter, atmospheric boundary layer, weather types, Gaussian plume model, low-level jet, recirculation, microscale, macroscale, mesoscale

## Abstract

Although emissions have a direct impact on air pollution, meteorological processes may influence inmission concentration, with the only way to control air pollution being through the rates emitted. This paper presents the close relationship between air pollution and meteorology following the scales of atmospheric motion. In macroscale, this review focuses on the synoptic pattern, since certain weather types are related to pollution episodes, with the determination of these weather types being the key point of these studies. The contrasting contribution of cold fronts is also presented, whilst mathematical models are seen to increase the analysis possibilities of pollution transport. In mesoscale, land–sea and mountain–valley breezes may reinforce certain pollution episodes, and recirculation processes are sometimes favoured by orographic features. The urban heat island is also considered, since the formation of mesovortices determines the entry of pollutants into the city. At the microscale, the influence of the boundary layer height and its evolution are evaluated; in particular, the contribution of the low-level jet to pollutant transport and dispersion. Local meteorological variables have a major influence on calculations with the Gaussian plume model, whilst some eddies are features exclusive to urban environments. Finally, the impact of air pollution on meteorology is briefly commented on.

## 1. Introduction

Air pollution is the subject of active research due to its marked impact on human life in a number of different environments, with regard to materials as well as living beings. Its impact on materials may have not only aesthetic but also economic implications. For example, the marginal costs of cleaning and repairing building façades due to air pollution have mainly been attributed to PM_10_ and SO_2_ [1], while in the plant environment, a key role is played by biomonitors. *Cupressus macrocarpa* leaves were recently used to evaluate the pollution load, and high values of anthropogenic elements, such as Cu and As, were observed near an industrial area [2]. Finally, the effects on human health have also been extensively investigated due to the global scope of the air pollution problem [3]. For instance, a positive and significant association between air pollutant concentration and the incidence of breast cancer has been described by Hwang et al. [4]. Moreover, the relationship between atmospheric levels of pollutants such as particulate matter, ozone, nitrogen dioxide, and sulphur dioxide and mortality causes has been reported in Portugal [5], with the increase in health expenditure due to air pollution being the economic implication of this issue [6].

Substances released into the atmosphere become pollutants when their concentrations exceed certain levels. These values may be reached not only by the increase in emissions but also under given meteorological conditions. The main difference between the roles of emissions and meteorological conditions is that emissions may be controlled when inmission levels are exceeded [7,8], whereas meteorological conditions cannot be controlled. Consequently, air pollution control strategies should take into account meteorological variables, since these will shape any such strategies to a certain degree. Kanawade et al. [9] analysed severe air pollution episodes in New Delhi, India, which were linked to stagnant atmospheric conditions. During the study period, low-level winds were below 8 ms^−1^, there was no precipitation, and 500 hPa wind speeds were below 13 ms^−1^. However, meteorology may also influence air pollution transport. Dong et al. [10] analysed the seasonal impact of meteorological variations on the air quality of the Beijing–Tianjin–Hebei region, China, which is a populated metropolitan region comparable to the Pearl River Delta or the Yangtze River Delta regions, where pollution transport plays a key role. One result of this study is the successful pollution control undertaken by joint regional controls. Moreover, the noticeable impact of meteorological conditions, up to 40% on regional transport over two seasons, i.e., spring and winter, suggested that regional air pollution needed to be controlled in both seasons.

The objective of this paper is to highlight the feedback between air pollution and meteorology. Consequently, the first goal of this paper is to simplify this topic, in which a range of disciplines, such as chemistry, physics, numerical modelling, biology, medicine, or even architecture, are involved. Moreover, this paper is not only aimed at pointing out the research lines that are currently active but also at highlighting possible future studies in order to attract potential researchers to a field that stands out thanks to its social impact and constant expansion.

Although the contribution of meteorological processes to air pollution events is usually assumed, it is frequently relegated to the background. Physical processes in the atmosphere are varied and include clear-sky surface solar radiation reduction by aerosols [11] or the study of their scattering angle [12], temperature inversions associated with air pollution and health effects [13], or analysis of electricity in the convective boundary layer [14]. Moreover, meteorological processes may have contrasting effects. Meteorological conditions sometimes determine air pollution situations, since they may aggravate pollution episodes, such as the recirculation of air parcel trajectories studied in Los Angeles [15]. However, certain meteorological conditions may also improve air quality, with the inverse relationship between particulate matter concentrations and the ventilation factor (the product of wind speed at 10 m and the mixed-layer height estimate) observed in Santiago City, Chile, being one noticeable example [16].

This study differs from previous analyses in the approach used, since it takes dynamic meteorology as a basis, due to the significant weight this subject carries in atmospheric analyses. Since energy in the atmosphere flows from large eddies to molecular processes, the structure of this paper follows the scale of atmospheric motions: macroscale, mesoscale, and microscale. Macroscale considers weather phenomena with a diameter above 1000 km and with a life of several days or even weeks, such as synoptic and tropical cyclones or fronts. Mesoscale corresponds to middle-sized atmospheric structures with a diameter between 2 and 1000 km and which last from hours to a couple of days, such as mountain waves or sea breezes. Finally, microscale is linked to processes of between less than a metre and a couple of kilometres and lasting from a few seconds to several minutes. Energy exchanges at the low atmosphere together with surface conditions determine atmospheric processes at this scale, such as tornadoes, dust devils, or plumes. This classification must be considered as the result of a simplification method to separate spatial and temporal ranges. However, in agreement with atmospheric evolution, processes may sometimes appear mixed, and their influence on air pollution needs to be evaluated.

Urban environments merit special consideration since large numbers of people live, work, meet, or spend time at these sites. Urban activities are sources of pollutants, and their emissions depend on city populations and facilities. Pollutant dispersion is conditioned by urban features, and the spread of cities may vary following cultural and geographical characteristics. Consequently, the impact of cities ranges from the micro to the mesoscale, and this review considers both possibilities.

A second key point of this study is its review of the influence of air pollution on meteorology, which has scarcely been analysed to date. This impact may be noticeable at a local scale, such as the effects observed by Saha et al. [17] during the eve of the Deepawali festival at Kolkata, India, when a massive firework display triggered a marked increase in particulate matter, the consequences of which were increased surface temperature and changes in the temperature vertical profile and diurnal pattern of relative humidity. However, the effects of air pollution may also be observed at a global scale. Wang et al. [18] simulated the long-range transport of anthropogenic aerosols from Asia, which moved on the north Pacific and led to the increase in cloud top height against preindustrial conditions, resulting in an invigoration of mid-latitude cyclones.

## 2. Macroscale

Two groups of papers investigate the influence of meteorology at this scale on the concentrations recorded. One considers the effect of the synoptic pattern, while the second focuses on pollutant transport, which is analysed using air parcel trajectory models.

### 2.1. Synoptic Pattern

Atmospheric flow may be classified by discrete circulation patterns in agreement with certain modes of atmospheric circulation. Both subjective and objective procedures may be used to achieve this goal. Dayan et al. [19] highlighted the importance of these classification methods in environmental phenomena such as air pollution, desert dust intrusions, and flash floods.

Dai et al. [20] used a classification method for regional particulate pollution days in Eastern China based on a subjective approach that employs only three patterns: equalised pressure (EQP), the advancing edge of a cold front (ACF), and the inverted trough of low pressure (INT). The seasonal features of these patterns were also considered to form eight types, three EQPs (in summer, autumn, and winter), two ACFs (in autumn and winter), and three INTs (in spring, autumn, and winter). Most of the pollution days appeared in winter, with the lowest number appearing in spring. Moreover, most of the pollution days were linked to the EQP, whereas the INT was the least frequent. The main weakness of subjective procedures is that they rely on the skill and experience of the classifier, since certain atmospheric flows may display features that are compatible with different patterns.

In contrast to this relatively simple and subjective classification, objective procedures are based on mathematical expressions that could be applied not only by users who have experience in atmospheric evolution but also, in some cases, by users who are beginning their career. One example is the Lamb–Jenkinson weather-type approach, which was considered by Li et al. [21] in their study into PM_2.5_ formation in North China. In this classification, the location of pressure centres was determined using gridded pressure data of a 16-point moveable region. Additionally, a small number of empirical rules was considered to classify the daily synoptic pattern as one of 26 weather types. As a result, three weather types were associated with high PM_2.5_ pollution levels. Another example of an objective classification is the obliquely rotated principal component analysis in T-mode, used by Mao et al. [22] to investigate air pollution over Wuhan in wintertime from 2014 to 2018. Three of five dominant synoptic patterns were linked to heavy pollution. A third example of objective weather synoptic classification is based on the self-organising map approach. Shu et al. [23] employed this procedure to analyse summertime ozone pollution over the Yangtze River Delta in China and found six predominant synoptic weather patterns: four ozone-polluted types and two ozone-clean types.

The synoptic pattern has a noticeable impact at the microscale level, with high-pressure systems being an example. They are linked to low wind speed, quite limited expansion of the boundary layer, and a reinforced inversion layer, which thereby increases pollutant concentration [24].

### 2.2. Cold Fronts

Yang et al. [25] analysed the weather systems associated with nine pollution episodes in Hong Kong. Four were linked with monsoons, two with tropical cyclones, one with a saddle-type pressure field, one with a warm high pressure, and one with a cold-front passage. Cold fronts are evolving structures that have a varied impact on air pollution. Jia et al. [26] analysed the “sawtooth cycles” in Beijing, although these features have also been observed in other mid-latitude areas, such as eastern North America, that are regularly crossed by the polar front. Sawtooths are linked with double fronts, where a weak cold front precedes a second and stronger cold front. Their period is irregular, frequently around five days, although some sawtooths extend up to a week and have been confused with regular, weekly cycles. The impact of this irregular synoptic cycle on particulate matter concentration is characterised by a slow increase over a few days followed by a sharp drop in just a few hours.

Rainfall associated with cold fronts determines the decrease of particulate concentrations [27]. Cold frontal passages usually favour atmospheric pollutant removal. Hu et al. [28] observed air pollution purification during the cold frontal passage associated with a strong cold-air outbreak, since surface wind speed and atmospheric boundary layer height increased. However, there is also evidence of quite different results. The formation and evolution of air pollution episodes is influenced by wind speeds and wind shears caused by nocturnal low-level jets and cold fronts [29]. Moreover, the chemical production of sulphate, nitrate, and ammonium may increase due to the uplift of air pollutant together with humidity, which facilitates the heterogeneous and aqueous-phase oxidation of precursors [30]. Another example is presented by Kang et al. [31], who observed that particulate matter may rise to the free troposphere from the North China Plain due to a cold frontal passage. These particles were transported by strong north-westerly frontal airflow and returned to the boundary layer at the Yangtze River Delta by synoptic subsidence under the high-pressure system that dominates once the frontal zone moves downstream. They concluded that cold fronts may be seen as potential carriers of atmospheric pollutants. Similarly, Zhou et al. [32] considered the influence of large-scale cold fronts and warm conveyor belts on the transport of continental aerosols located near the surface mixed with aerosols from the upper planetary boundary layer and middle troposphere. Finally, Song et al. [33] analysed a dust storm event in Northern China in early May 2017 and concluded that the front’s activity was associated with increased wind speed and dust emission.

### 2.3. Pollution Transport

Air pollution transport has an impact not only on people’s health but also on political relationships between countries who share common borders, with particulate matter transport from Poland to the Czech Republic being one notable example when the wind direction may be established, which occurs in around 50% of cases. This is true despite the prevailing flow being from the Czech Republic to Poland, which is typical of northeast Moravia due to the orographic influence of the Moravian Gate, and though emissions from both countries contribute equally with low wind speeds, since wind direction frequently changes under such conditions. Most of the pollution was attributed to days with unclear airflow or with a significant wind change [34]. Another example is the pollution from China that affects South Korea’s air quality with a seasonal evolution [35]. In this analysis, agricultural burning and coal-fired heating were noticeable in summer and winter, respectively, while dust storms from the deserts impacted in spring.

Different models have been developed to explore air parcel trajectories. The HYSPLIT (Hybrid Single-Particle Lagrangian Integrated Trajectory) model has been widely used. It was developed by the NOAA (National Oceanic and Atmospheric Administration) and, since it was first described by Draxler and Hess [36], it has been regularly updated [37]. However, other models have also gained certain relevance. The FLEXPART (FLEXible PARTicle dispersion model) may simulate long-range transport, turbulent diffusion, deposition, or linear chemistry. Since it first appeared in the mid-1990s [38], the model has also improved its performance, physical–chemical parameterisations, input and output formats, or available software [39]. Another example is the METEX (METeorological data EXplorer) model [40], whose main feature is its easy application, since only the coordinates of the site of interest are required and air parcel trajectories may be systematically calculated.

Determining air parcel trajectories is essential in terms of knowing which locations are affected by hazardous substances that have been accidentally released, such as radionuclides [41]. Air parcel trajectory models may be a necessary tool to determine the atmospheric corridors that control air flow. However, a large number of air parcel trajectories need be considered if any firm conclusions are to be reached, since handling isolated trajectories may not prove to be easy. Procedures for grouping trajectories, such as clustering analysis, are required and possible applications vary. Salmabadi et al. [42] employed the total spatial variance method to identify four main dust corridors affecting Ahvaz, Iran. Three of these, which had westerly or north-westerly orientations, were associated with the Shamal winds and were particularly active in summer. Another corridor, with a southerly orientation, was linked to a prefrontal dust-storm mechanism and was active in spring and winter. Rana et al. [43] considered the “angle distance matrix procedure” to form six trajectory groups and obtained high particulate matter concentration when the air parcels followed the route from the Middle East through the Himalayan valley to Dhaka, Bangladesh, during the dry season. Moreover, theoretical trajectory calculations must be combined with measurements and satellite imagery in order to obtain a precise knowledge of potential sources and transport times [44]. Trajectory calculations are the basis of certain useful calculations, such as the potential source contribution function and concentration-weighted trajectories [45]. In both procedures, the study region is divided into cells and the number of trajectories calculated must be high enough to obtain reliable results. The potential source contribution function considers the relationship between trajectory endpoints above a given concentration level and the total endpoints in each cell, whereas concentration-weighted trajectories calculate the average weighted concentration in each cell.

## 3. Mesoscale

Typical mesoscale processes are breezes induced by land–sea interactions. Orographic features condition the transport and dispersion of air pollution, and this interaction is the subject of ongoing research [46]. Moreover, air parcel recirculation and urban heat island effects also lie within this scale.

### 3.1. Breezes

Land–sea breezes are formed at the interface of surfaces with contrasting features. At these sites, transport of particulate matter and its composition reveal its origin. Batchvarova et al. [47] measured aerosol concentration at Ahtopol (Bulgaria) and found a lower number of coarse particles for marine air masses. Another example is the Pearl River Delta, where interactions of synoptic winds and mesoscale breezes determined weak winds and air pollution accumulation, favouring intense photochemical reactions and the formation of an ozone “pool” [48]. Similarly, in Manila, Philippines, strong sea breezes disperse fine particulate matter inland during the day in spring, although breezes are the outflow path of these aerosols during the night. However, stagnant air determines high accumulated particulate concentration in autumn when the sea breeze is weaker [49]. Dasari et al. [50] indicated that differential heating between land and sea caused strong breezes that led to diurnal dispersion and distribution of pollutants in Neom, Saudi Arabia.

Several examples illustrate the role of orographic features in pollutant transport and dispersion. Bei et al. [51] analysed the influence of mountain–valley breeze circulations in the Guanzhong basin, China, and found that this breeze determines a convergence zone in the basin where pollutants return from the mountain and concentrations increase in the evening. Alternatively, different indicators may be used to characterise the relief effect on atmospheric flow. Lai and Lin [52] analysed wind direction, the Froude number, and the pitching angle of the surface level among other variables to investigate air pollution events in Taiwan. They considered ten categories of air pollution events following the airflow and the location of the air pollution event. Moreover, the Froude number was below 0.25 in most of these events, revealing the high stability of the airflow, which tends to split around the mountains. Another noticeable effect induced by the relief was described by Quan et al. [53], who observed that a vertical vortex elevated ground pollutants at an altitude between 1.4 and 1.7 km where an elevated pollutant layer is formed and transported to Beijing by the southerly wind.

### 3.2. Recirculation Processes

Allwine and Whiteman [54] proposed integral quantities to describe stagnation, recirculation, and ventilation to characterise airflow at specific sites. Stagnation conditions may favour high pollutant concentrations. However, recirculation is quantified by the recirculation factor where 0 corresponds to straight-line transport without recirculation, and 1 is reached when the air parcel returns to its origin without net transport. In this latter case, concentrations may change when pollutants are affected by chemical reactions. One example is recirculation formed by sea breezes combined with upslope winds in the western Mediterranean basin during summer, and which may be considered as natural-photochemical reactors, since nitrogen oxides and precursors are transformed into oxidants, such as ozone, under strong insolation with residence times in the order of days [55]. Recirculation has also been observed in the Hong Kong area, another coastal site, where ozone pollution may be aggravated by this process [56]. Dimitriou and Kassomenos [57] analysed the impact of Saharan dust intrusions on continental Greece and concluded that certain infrequent events observed in August–September were due to dust recirculation by the Etesian winds linked to the extension of the Azores anticyclone in continental Europe. Since recirculation is a kind of eddy, orographic features, such as valleys, favour these processes. Quimbayo-Duarte et al. [58] analysed the air flow in a valley with two sections and observed recirculations when the air from the slopes converges at the valley floor and flows to the narrow section in the up-valley direction.

Another example of recirculation is the Fresno eddy [59], which is a cyclonic vortex formed during the night in the San Joaquin Valley of California. During the daytime, winds mainly enter through the San Francisco Bay area and transport precursors to the valley where ozone is formed. The air rises into the mountains at the southern end and leaves the valley. However, during the night-time, the air cannot leave the valley due to the cool wind drainage from the mountains and so returns north in a circular flow pattern, known as the Fresno eddy. Pollutants are forced to return to urban areas where more precursors are added [60]. Observations indicate that this eddy dominates ozone production in the central part of this valley and triggers many ozone exceedances if it is strong on apparently ventilated days [61].

### 3.3. Urban Heat Island

The features as well as the extension of certain urban structures can modify both thermal and mechanical turbulences. Absorption and emission of radiation by urban surfaces in urban environments differs from the same processes when they occur in rural surfaces. Moreover, the roughness determined by buildings is also greater. The urban heat island may enhance the existing vorticity, which produces direct thermal circulation, causing surface mesovortices. Mainhart et al. [62] observed that ozone formed outside the Saint Louis metropolitan area returned to the city due to such mesovortices.

The beneficial effect of urban heat island mitigation by urban cooling strategies on air quality is assumed. Green roofs and living walls, for example, are useful for removing air pollutants [63]. However, competing feedbacks should be considered since green and cool roofs reduce wind speed and vertical mixing during the day, which might lead to air stagnation and air pollution situations [64]. Similarly, Henao et al. [65] indicated that air pollution transport mechanisms in urban valleys could be altered by urban heat island mitigation and that air quality could worsen. In fact, Li et al. [66] presented the “urban aerosol pollution island”, which is negatively correlated with the urban heat island in summer. Moreover, mitigation of the urban heat island by an increasing albedo weakened turbulent mixing, made the planetary boundary layer height decrease, and led to a stronger urban aerosol pollution island. Consequently, a balance needs to be found between urban heat island mitigation and its impact on air quality.

Droste et al. [67] introduced the “urban wind island effect” since, under certain atmospheric conditions, wind speed in the city may be higher than that corresponding to rural environments. This effect would be observed in the afternoon and would be due to the contrast between the city and the countryside in the atmospheric boundary layer growth, surface roughness, and ageostrophic wind. However, this effect has only been suggested and needs to be verified prior to gauging its influence on air pollution.

## 4. Microscale

At the shortest spatial scale, pollutant concentration is affected by the atmospheric boundary layer height. However, determining its depth may not prove to be easy, and the issue has been avoided in various studies where concentrations are related with meteorological variables measured at a local scale.

### 4.1. Atmospheric Boundary Layer

Su et al. [68] found strong interactions between the planetary boundary layer height and particulate matter concentration in winter, when the planetary boundary layer height is shallow and particulate matter concentration is high. Correlations between the planetary boundary layer height and particulate concentrations tend to be negative in general. However, the magnitude, significance, or sign of these correlations depend on the location, season, and meteorological conditions. Results indicate that this relationship is weak over clean regions but nonlinear over polluted regions.

The development of a thermal internal boundary layer at coastal sites is linked with pollution episodes. Wei et al. [69] obtained the boundary layer height with a ceilometer at Qinhuangdao, North China. Determining the thermal internal boundary layer was based on wind direction, when the wind blew from the sea, and boundary layer height. The boundary layer was thinner with a thermal internal boundary layer. If this layer was observed, particulate concentrations increased when the boundary layer height rose, since the dilution effect of the breeze is weak, and the boundary layer height was small. However, if the thermal internal boundary layer was not formed, particulate concentrations fell when the boundary layer height increased.

The atmospheric boundary layer, which develops during the day, evolves to a stable nocturnal boundary layer due to the radiative cooling of the ground and is topped by a residual layer, which is a reservoir of pollutants from daytime emissions and photochemical production. Observations indicate a wind speed maximum, known as low-level jet, near the top of the nocturnal boundary layer. This nocturnal jet may favour mixing between the two layers despite stable stratification.

The influence of low-level jets on pollutant concentrations varies. These jets may transport pollutants trapped in the residual layer and trigger episodes when mixed at the surface by convective turbulence after sunrise. One example of this process is presented by Sullivan et al. [70], who reported an ozone episode in the Baltimore–Washington DC urban corridor in June 2015, with concentrations between 70 and 100 ppb at around 1 km above the surface in the nocturnal residual layer. These concentrations were transported by the nocturnal low-level jet, and both the simulations and the measurements indicated high “next-day” ozone concentrations at sites along the southern New England region. A similar case is presented by Li et al. [71], where pollutants from the North China Plain were transported by the nocturnal low-level jet to Shenyang. Caputi et al. [72] suggested that the ozone of the residual layer is actively mixed in the nocturnal boundary layer when the low-level jet is strong. Under these conditions, ozone dry deposition causes low concentrations the following day. However, ozone is retained by the residual layer when the low-level jet is weak since the residual layer is more decoupled. In this situation, fumigation processes the following morning would determine high ozone concentrations near the ground. Wei et al. [73] also suggested an abrupt reduction in particulate matter concentration and an improvement in air quality due to enhanced vertical mixing linked to the low-level jets formed at the end of the pollution periods.

Wind speed and vertical turbulence control air pollution during the day. However, vertical turbulence is weak at night and vertical mixing is controlled by large-scale turbulence eddies whose increased number, due to low wind speed, might lead to a fall in concentration near the ground [74].

### 4.2. Local Meteorological Variables

Specialised devices are not usually available to most researchers. In these situations, some analyses relate pollutant concentrations with variables provided by meteorological stations.

Chen et al. [75] observed that temperature and air pressure had a marked influence on ozone concentrations in Beijing, although meteorological influence was weak in summer, since emissions of volatile organic compounds and nitrogen oxides increased in this season and led to ozone pollution episodes. The close relationship between particulate matter and meteorological variables has been the subject of several analyses. For instance, Meng et al. [76] concluded that the planetary boundary layer height and temperature difference of the inversion layer and its depth are variables that impact particulate matter concentrations. This analysis presented the ranges of these meteorological variables, where the correlation with air pollution was observed. In this line, certain thresholds of the meteorological variables should be established, since the relationship with air pollution may be the opposite. Wang et al. [77] considered 27 cities in China and studied surface solar irradiance and wind speed. Both variables displayed a similar trend between 1961 and 2011. The authors found that winds below 2.5 ms^−1^ disperse air pollutants and enhance solar surface irradiance. However, winds above 3.5 ms^−1^ enhance aerosol concentration, perhaps from dust storms, and surface solar irradiance was seen to be attenuated. Between the two thresholds, no relationship between wind speed and surface solar radiation could be established.

Local meteorology may have a marked influence when a hazardous substance is accidentally released or when an industrial facility is close to a population centre, and various mathematical models have been developed to investigate air pollutant dispersion. The Gaussian model is a classical formulation of plumes that presents a simple structure and allows multiple simulation possibilities. Moreover, it may easily be extended to more complex configurations of sources. Şahin and Ali [78] determined the emergency planning zones around two nuclear power plants using this model, and Rohmah and Nurokhim [79] simulated I^131^ dispersion around a nuclear facility. Similarly, Maués et al. [80] applied this model to evaluate the air quality of Volta Redonda, Brazil, where a large steel plant is located, and reported concentration curves around the plant showing the plume impact. Dispersion coefficients play a key role in this model. Although their calculation relies on empirical schemes, the parameterisations used may give unequal results and should be compared. Mao et al. [81] considered four dispersion schemes applied to the Prairie Grass experiments in 1956. They evaluated the performance of these schemes following the stability conditions and selected the Chinese National Standard from the results obtained for the different stability classes.

### 4.3. Urban Micrometeorology

Eddy formation is favoured by the rough elements and varied covers present in cities. Han et al. [82] compared the air quality between urban and rural areas in 35 major Chinese metropolitan regions. Although urban pollution is usually more marked than rural pollution in summer, both pollutions are noticeable in winter, with rural pollution being even stronger than urban pollution in certain cities. The authors found that the key factors for the longer spatial inhomogeneity in summer versus winter were emissions, urban structures, meteorology, topography, and humidity. At a smaller, daily timescale, Choi et al. [83] observed in Los Angeles that limited dispersion capacity and the relatively stable surface layer in the morning led to higher ultrafine particle concentrations than in the afternoon. In this period, wind speeds were higher and turbulence was stronger, with vertical turbulence intensity being the most effective factor controlling ultrafine particle concentrations. The effect of tall buildings on pollutant dispersion was studied by Aristodemou et al. [84], who found a concentration increase at high floors, whereas dispersion was favoured by tall buildings at low floors.

## 5. Influence of Air Pollution on Meteorology

Although the spread and intensity of atmospheric processes make them insensitive to external influences, the strength of air pollution might condition an area’s weather or even its climate.

Wang et al. [85] studied the effect of aerosols on radiation. Surface temperature is seen to be reduced by solar radiation reduction and the temperature increase of the upper layer by solar radiation absorption or backscattering. As a result, atmospheric stratification becomes more stable. In fact, simulations with increasing aerosol optical depth have revealed a transition from unstable to stable stratification at rural sites, to weakly stable at suburban sites and to less unstable or neutral stratification at urban sites. Consequently, energy transfer from the surface is reduced, as is the boundary layer height. This impact of aerosol pollution on meteorology has been reported in numerous analyses. Nguyen et al. [86] also observed a decrease in shortwave radiation, temperature, planetary boundary layer height, and wind speed. Visibility degradation in Eastern Thailand has been studied by Aman et al. [87], who observed a marked seasonality as well as a relationship with wind direction. Moreover, this visibility degradation was linked to air pollution and meteorological conditions, such as small wet scavenging, low mixing height, and a high recirculation factor.

Evidence concerning the relationship between aerosols and precipitations is conflicting. Rosenfeld and Woodley [88] indicated that the number of cloud condensation nuclei in polluted air is over ten times higher than this number for the same volume of clean air. Consequently, under air pollution conditions, small droplets would be formed, which could not easily grow to reach precipitation size. Jirak and Cotton [89] observed this effect at elevated sites west of Denver and Colorado Springs, Colorado. However, Rosenfeld et al. [90] have explained both the decrease and the increase in rainfall by air pollution from a combination of radiative effects on surface heating and microphysical effects on invigorating the convection. Recently, Yang et al. [91] concluded that tropical cyclone precipitation was invigorated by aerosols over the Chinese mainland from 1980 to 2014. Moreover, light rain presented a decreasing trend against time, whereas the trend for heavy rains was seen to be increasing. In contrast, post-monsoon precipitation reduction in South Asia between 2004 and 2014 was explained by two factors. The first was aerosol loading, which triggered surface cooling, and the second was the temperature decrease by evaporation around irrigation hotspots [92].

## 6. Conclusions

Meteorological conditions have a direct influence on emission control in air pollution events. Although atmospheric processes are varied and frequently mixed, one or a few sometimes prevail and a direct relationship with high pollutant concentrations may be established.

At the synoptic scale, objective procedures provide for a systematic classification of weather types that can be linked to pollution events. However, pressure centres, which are associated with certain air mass features, move slowly and their fronts sweep the low atmosphere, with contrasting effects on air pollution. Air parcel trajectory models use meteorological observations and are a useful tool to investigate the sources, paths, and destinations of air pollution.

Air flow may be disturbed by coasts, mountains, or big cities. These features may determine horizontal or vertical vortices where pollutants may be trapped. Recirculation processes have been the subject of some studies, and their analysis continues to offer a promising line of research. Moreover, the contrast between large cities and their surrounding areas is evolving rapidly, and the corresponding changes in meteorological aspects or consequences in pollutant transport and dispersion are fields that remain open to study.

At the lowest scale, the boundary layer height is inversely correlated with pollutant concentrations. Boundary layer evolution is closely related with air pollution since a polluted residual layer may be formed at night and the low-level jet favours both pollutant transport and dispersion. The relationship between concentrations and the usual meteorological variables is common in air pollution studies. Cities are particular sites that favour turbulence and where the low atmosphere is more unstable than at rural sites. The Gaussian plume model enables pollutant concentration to be calculated and is especially useful when dealing with the release of hazardous substances. Moreover, this model may be easily adapted to greater distances and varied conditions such as trapped plumes, fumigation, or orographic features. At this scale, thresholds of meteorological variables should be investigated following air pollution levels.

Finally, the impact of air pollution on meteorology should not be excluded, with radiation absorption by aerosols being one example. The relationship between aerosols and precipitation depends on the balance between microphysical and radiative effects, and the conditions linked to this balance should be analysed.

Consequently, the examples presented in this study demonstrate that the relationship between meteorology and air pollution merits in-depth enquiry, and air pollution control strategies can no doubt benefit from this research. Although these studies may appear to be local analyses, ascertaining common features remains open to further research in order to gain insights into the feedback between meteorology and air pollution.
